# Retrospection dropout bare-bones particle swarm optimization for feature-based brain tumor classification in MRI images

**DOI:** 10.3389/fonc.2026.1738658

**Published:** 2026-04-13

**Authors:** Wenting Chen, Tiezhu Shi, Jia Guo, Ling Fu

**Affiliations:** 1 School of Biomedical Engineering, State Key Laboratory of Digital Medical Engineering, Key Laboratory of Biomedical Engineering of Hainan Province, Hainan University, Haikou, China; 2 Hematology Department of Hainan General Hospital, Hainan, Haikou, China; 3 State Key Laboratory of Subtropical Building and Urban Science & Key Laboratory for Geo-Environmental Monitoring of Coastal Zone of the National Administration of Surveying, Shenzhen University, Shenzhen, China; 4 Hubei Key Laboratory of Digital Finance Innovation, Hubei University of Economics, Wuhan, China; 5 School of Information Engineering, Hubei University of Economics, Wuhan, China; 6 Graduate School of Computer and Information Sciences, Hosei University, Tokyo, Japan

**Keywords:** bare-bones, brain tumor, MRI images, particle swarm optimization, retrospection dropout

## Abstract

Brain cancer remains a critical global health challenge, where early and accurate diagnosis remains a critical challenge in clinical practice. Current supervised learning methods for tumor classification face substantial limitations due to their dependence on large labeled datasets requiring costly pixel-level annotations, susceptibility to annotation biases, and poor generalization across diverse populations. To address these challenges, this paper proposes Retrospection Dropout Bare-Bones Particle Swarm Optimization (RDBPSO), a novel feature-based classification framework that requires only image-level class labels without the need for pixel-level annotation or manual segmentation masks. The proposed RDBPSO introduces two key innovations: (1) a retrospection mechanism that maintains dual-layer memory structures (optimal and sub-optimal solutions) to enhance particle diversity and prevent premature convergence, and (2) a dropout strategy that reduces computational complexity through intelligent particle interaction sampling. Extensive experiments on an 800-image brain MRI dataset demonstrate RDBPSO’s superior performance. The proposed method achieves 90.12% classification accuracy, outperforming standard PSO (89.25%), GMM (77.50%), and K-means (72.75%), while delivering robust clustering quality with an ARI of 0.6436, NMI of 0.5511, and FMI of 0.8229. These results demonstrate the algorithmic promise of RDBPSO as an annotation-efficient framework for brain tumor MRI classification, warranting further investigation on more diverse and clinically representative datasets.

## 1 Introduction

Brain cancer represents one of the most formidable health threats globally, ranking among the leading causes of cancer-related mortality across all demographics Kader et al. (1). The disease’s heterogeneous nature, characterized by diverse tumor types and varying clinical presentations, poses significant challenges for accurate diagnosis and effective treatment planning. Early detection remains the cornerstone of successful brain cancer management, as it dramatically improves survival rates and treatment outcomes Gumaei et al. (2).

The identification and classification of brain cancer present multifaceted challenges that complicate diagnostic processes Irmak (3). The disease exhibits considerable phenotypic diversity, with tumors displaying varying degrees of aggressiveness, cellular composition, and genetic profiles. Traditional diagnostic approaches often rely on subjective interpretations of medical imaging and histopathological examinations, leading to potential inter-observer variability and diagnostic inconsistencies Akter et al. (4). Furthermore, the subtle differences between benign and malignant lesions, particularly in early-stage cases, can result in misclassification and delayed treatment initiation. The complexity is further amplified by the need to distinguish between different brain cancer subtypes, each requiring tailored therapeutic interventions Sharif et al. (5). Moreover, existing deep learning approaches often suffer from high computational complexity and extensive parameter requirements, leading to prolonged execution times and demanding system specifications Deepak and Sarath (6).

Conventional supervised learning methods for brain cancer classification, while demonstrating promising results, suffer from several critical limitations that hinder their widespread clinical implementation Poongu-zhali et al. (7). The limitations of supervised approaches underscore the critical necessity for complex classification methodologies in brain cancer diagnosis Ali et al. (8)Joshi and Aziz (9).

While pixel-level annotation methods such as segmentation masks enable high-precision tumor localization, the acquisition of such fine-grained labels is prohibitively expensive and time-consuming. One promising strategy to alleviate this annotation burden is to leverage synthetic data generation techniques to augment existing labeled datasets; however, current generative approaches remain limited in their generalizability across diverse imaging protocols and scanner configurations Abbasi et al. (10)Ding et al. (11).

In response to these challenges, this study proposes a novel brain tumor classification framework that requires only image-level class labels, without the need for pixel-level annotation or manual segmentation. The main contributions and innovations of this research are threefold: (1) This study introduces the Retrospection Dropout Bare-Bones Particle Swarm Optimization (RDBPSO) algorithm, a feature-based classification approach that achieves high-precision brain tumor classification using only image-level labels. Unlike conventional supervised methods that depend on costly pixel-level annotations such as segmentation masks, the proposed RDBPSO framework operates on handcrafted image features optimized by swarm intelligence, substantially reducing the annotation burden while maintaining competitive classification accuracy. (2) The RDBPSO algorithm incorporates an innovative retrospection strategy that endows each particle with a sophisticated dual-layer memory structure. This mechanism maintains both optimal and sub-optimal historical solutions, enabling particles to retain valuable information from previous exploration phases. The dual-layer memory architecture significantly enhances the algorithm’s robustness by preventing premature convergence to local optima and facilitating more comprehensive exploration of the solution space, leading to more reliable and stable classification outcomes. (3) To address the computational complexity inherent in swarm-based optimization, this work introduces a strategic dropout mechanism that intelligently reduces computational overhead without compromising solution quality. The dropout strategy randomly selects a subset of particle interaction combinations during each iteration, effectively balancing exploration thoroughness with computational efficiency, achieving a 50% reduction in function evaluations per iteration and making the proposed method particularly suitable for large-scale medical image analysis research.

The remainder of this paper is organized as follows. Section 2 reviews the existing literature. Section 3 elaborates on the experimental datasets and the proposed RDBPSO algorithm, encompassing the dual-layer memory architecture, the dropout strategy, and the high-precision classification framework. Section 4 presents the experimental pipeline along with comparative results against representative benchmark clustering algorithms. Section 5 discusses the key findings, algorithmic advantages, and inherent limitations of the proposed method. Finally, Section 6 concludes the paper and identifies promising directions for future research.

## 2 Related works

Brain tumors represent a leading cause of cancer-related mortality worldwide, affecting both pediatric and adult populations. Accurate classification of brain tumor grades, particularly the distinction between low-grade and high-grade gliomas at early stages, is crucial for successful prognosis and the development of effective treatment strategies. However, precise computerized classification of brain tumors remains challenging due to variations in tumor size, shape, location, and inherent limitations within the medical domain Yaqub et al. (12)Kang et al. (13).

Recent advances in deep learning have facilitated the development of artificial intelligence-powered brain tumor grading systems capable of assisting radiologists in rapid medical image interpretation. Convolutional Neural Networks (CNNs), as one of the most prominent deep learning methodologies for visual learning and image classification tasks, have been extensively applied in brain tumor classification. Rasheed et al. (14) Kurdi et al. (15) Dhaniya and Umamaheswari (16) proposed novel learning-based algorithms for brain tumor classification. This characteristic is particularly valuable in clinical scenarios where labeled data is limited or unavailable Zeng et al. (17). Additionally, particle swarm optimization-based methods demonstrate enhanced adaptability to novel data patterns and improved generalizability across different patient populations and clinical settings Abdi et al. (18).

However, a primary limitation of existing technologies lies in their complexity, characterized by substantial parameter counts that contribute to prolonged execution times and demanding system specifications for implementation. To address these computational complexity issues, Kesav et al. Kesav and Jibukumar (19) proposed a novel architecture for brain tumor classification and tumor type object detection utilizing RCNN techniques. They initially employed a two-channel CNN, a low-complexity architecture, to distinguish between glioma and healthy tumor MRI samples, achieving an accuracy of 98.21%. Additionally, researchers have explored various optimization strategies, with Pradeep et al. Pradeep et al. (20) developing an accelerated particle swarm optimization-based artificial neural network model for improved tumor classification efficiency. Despite these advances, conventional machine learning approaches remain constrained by limitations including extensive labeled data requirements, limited generalizability, and substantial computational resource demands.

To overcome the limitations of single-modality approaches, researchers have begun exploring multimodal and semi-supervised learning techniques. Ge et al. Ge et al. (21) addressed glioma classification challenges using four MRI scan modalities: T1-weighted MRI, T1-weighted MRI with contrast enhancement, T2-weighted MRI, and FLAIR sequences. Recognizing that numerous available glioma datasets contain unlabeled brain scans and are of moderate size, they proposed leveraging deep semi-supervised learning to fully exploit unlabeled data.

Similarly, Hao et al. Hao et al. (22) introduced a transfer learning-based active learning framework designed to reduce annotation costs while maintaining stability and robustness of model performance for brain tumor classification. In their retrospective investigation, they adopted a 2D slice-based approach to train and fine-tune models on MRI datasets comprising 203 training patients and 66 validation patients. Recent studies have also explored advanced preprocessing and feature extraction techniques to enhance classification performance. Isuwa et al. Isuwa et al. (23) conducted a comprehensive survey on swarm intelligence algorithms for optimizing cancer gene selection, highlighting the potential of these methods in biomedical applications. Similarly, Mohan et al. Mohan et al. (24) developed a handcrafted deep-feature-based approach combining ResNet with chicken swarm optimization for brain tumor detection and classification.

With the growing demand for intelligent learning methodologies, the application of evolutionary computation techniques in medical image analysis has garnered increasing attention. Traditional Particle Swarm Optimization (PSO) algorithms simulate flocking behavior to search for optimal solutions; however, they are susceptible to local optima entrapment in complex multi-modal optimization problems. Recent developments in PSO variants have shown promising results in medical image analysis applications. Guo et al. Guo et al. (25) introduced a twinning bare bones PSO algorithm that enhances local minimum escaping ability, while Deepa et al. Deepa et al. (26) proposed a modified PSO segmentation approach with ensemble classification. Furthermore, advanced evolutionary algorithms such as the hermit crab optimization algorithm Guo et al. (27) and paired barracuda swarm optimization Guo et al. (28) have demonstrated superior performance in high-dimensional optimization problems, indicating their potential applicability to medical image classification tasks.

The latest developments in evolutionary computation have introduced sophisticated memory mechanisms and optimization strategies. Guo et al. Guo et al. (29) presented a bare-bones particle swarm optimization with crossed memory for enhanced global optimization, while their subsequent work Guo et al. (30) introduced a novel snow leopard optimization algorithm specifically designed for high-dimensional feature selection problems. Recent advances have also extended to complex scene analysis applications, with Shi et al. Shi et al. (31) developing visual image-based complexity perception methods using paired bare bone particle swarm clustering. Most recently, Guo et al. Guo et al. (32) proposed a deep backtracking bare-bones particle swarm optimization algorithm that achieved remarkable performance in high-dimensional nonlinear function optimization, demonstrating the continued evolution and refinement of these methodologies.

Recent applications have also demonstrated the versatility of evolutionary approaches beyond traditional classification tasks. Guo et al. Guo et al. (33) developed a novel breast cancer image classification model based on multiscale texture analysis and dynamic learning strategies, showcasing the potential for evolutionary methods in medical imaging applications. These developments collectively indicate the promising trajectory of evolutionary computation techniques in addressing complex medical image analysis challenges while minimizing the dependency on extensive labeled datasets.

## 3 Materials and methods

### 3.1 Experimental data

This brain medical imaging dataset comprises 800 grayscale images equally distributed between healthy (408 samples, 51%) and tumor (392 samples, 49%) categories, providing an ideally balanced classification scenario. The images have been preprocessed to a uniform resolution of 256×256 pixels and stored in JPG format. A comprehensive set of 16 features has been extracted from each image, encompassing five GLCM-based texture descriptors (contrast, correlation, energy, homogeneity, and entropy), six first-order statistical measures (mean, standard deviation, skewness, kurtosis, and percentile values q10 and q90), four wavelet transform energy coefficients derived from multi-resolution sub-band decomposition, and edge density computed from intensity gradient analysis.

The experimental data in this study are all from public datasets (License CC0: Public Domain) and do not involve any privacy, ethical and copyright issues. Datasets can be accesses from Kaggle: https://www.kaggle.com/datasets/hamzahabib47/brain-cancer-detection-mri-images.

### 3.2 Retrospection Dropout Bare-Bones Particle Swarm Optimization

The Retrospection Dropout Bare-Bones Particle Swarm Optimization (RDBPSO) algorithm represents a sophisticated evolution of traditional particle swarm optimization techniques, specifically designed to address the challenges of high-precision classification in high-dimensional medical data. RDBPSO integrates three core components: the foundational bare-bones particle swarm optimization framework, an innovative retrospection mechanism with dual-layer memory architecture, and a strategic dropout approach for computational efficiency enhancement.

The algorithm operates by maintaining a population of particles that explore the solution space through collaborative search mechanisms. Unlike conventional PSO variants that rely on velocity-based updates, RDBPSO employs Gaussian distribution-based position updates while incorporating historical memory structures and selective interaction strategies to achieve superior optimization performance.

#### 3.2.1 Particle representation and initialization

In RDBPSO, each particle *i* in the swarm is characterized by multiple memory layers:

- Current position: $x_{i}^{t}\in {\mathbb{R}}^{d}$.
- Personal best memory: $y_{i}^{t}\in {\mathbb{R}}^{d}$ with fitness $f(y_{i}^{t})$.
- Personal second-best memory $y_{i,\text{second}}^{t}\in {\mathbb{R}}^{d}$ with fitness $f(y_{i,\text{second}}^{t})$.

The swarm maintains global memory structures:

- Global best position: $g_{\text{best}}^{t}$ with fitness $f(g_{\text{best}}^{t})$.
- Global second-best position: $g_{\text{second}}^{t}$ with fitness $f(g_{\text{second}}^{t})$.

Initialization Process.

The initialization process ensures robust dual-layer memory establishment:

For each particle *i* = 1,2*,…,N*:

1. Generate two random positions: $\;pos_{1}^{i},pos_{2}^{i}∼U(lb,ub)$.
2. Evaluate fitness: $\;f_{1}=f(pos_{1}^{i})$, $f_{2}=f(pos_{2}^{i})$.
3. Assign memories based on fitness comparison.

Details are shown in Equation 1.

(1) $$\left\{ \begin{array}{ll} y_{i}=pos_{1}^{i},\;y_{i,\text{second}}=pos_{2}^{i}, & \text{if}\;f(pos_{1}^{i})\leq f(pos_{2}^{i})\\ y_{i}=pos_{2}^{i},\;y_{i,\text{second}}=pos_{1}^{i}, & \text{if}\;f(pos_{1}^{i})>f(pos_{2}^{i}) \end{array}\right.$$

where $y_{i}$ is the best position of particle *i*, $y_{i,\text{second}}$ is the second-best position of particle *i*, $pos_{1}^{i}$ and $pos_{2}^{i}$ are two random positions generated for particle *i*, and f(·) is the objective function to be minimized.

#### 3.2.2 Dual-layer memory architecture

The retrospection mechanism fundamentally enhances the algorithm’s memory capacity by maintaining multiple historical solutions for each particle. This dual-layer architecture serves several critical functions:

Primary Memory Layer: Stores the best solution discovered by each particle, representing the optimal performance achieved thus far.

Secondary Memory Layer: Maintains the second-best solution, preserving valuable alternative solutions that might become relevant as the search progresses.

#### 3.2.3 Memory update strategy

The memory update process follows a hierarchical structure, details are shown in Equation 2.

(2) $$(y_{i},y_{i,\text{second}})=\{\begin{array}{ll} (x_{\text{new}},y_{i}), & \text{if}\;f_{\text{new}}<f(y_{i})\\ (y_{i},x_{\text{new}}), & \text{if}\;f(y_{i})\leq f_{\text{new}}<f(y_{i,\text{second}})\\ (y_{i},y_{i,\text{second}}), & \text{otherwise} \end{array}$$

where $x_{\text{new}}$ is the newly generated candidate solution with fitness value $f_{\text{new}}=f(x_{\text{new}})$, $y_{i}$ is the current personal best position of particle *i* with fitness $f(y_{i})$, and $y_{i,\text{second}}$ is the second-best position with fitness $f(y_{i,\text{second}})$.

#### 3.2.4 Dropout strategy

Two random particles are paired into one combination; the one with better fitness is the leader, and the other is the worker. The dropout strategy addresses computational complexity by intelligently selecting particle interaction combinations. For each particle pair processing:

Better Particle Combinations (4 total possibilities):

-
$\left(g_{\text{best}},y_{\text{better}}\right)$: Global best with personal best.
-
$\left(g_{\text{second}},y_{\text{better}}\right)$: Global second-best with personal best.
-
$\left(g_{\text{best}},y_{\text{better},\text{second}}\right)$: Global best with personal second-best.
-
$\left(g_{\text{second}},y_{\text{better},\text{second}}\right)$: Global second-best with personal second-best.

Worse Particle Combinations (4 total possibilities):

-
$\left(y_{\text{better}},y_{\text{worse}}\right)$: Better particle’s best with worse particle’s best.
-
$\left(y_{\text{better},\text{second}},y_{\text{worse}}\right)$: Better particle’s second-best with worse particle’s best.
-
$\left(y_{\text{better}},y_{\text{worse},\text{second}}\right)$: Better particle’s best with worse particle’s second-best.
-
$\left(y_{\text{better},\text{second}},y_{\text{worse},\text{second}}\right)$: Both particles’ second-best memories.

For each selected combination (*p*_1_*,p*_2_), new candidates are generated using Equation 3.

(3) $$x_{\text{candidate}}=N(\frac{p_{1}+p_{2}}{2},|p_{1}-p_{2}|)$$

where $x_{\text{candidate}}$ is the newly generated candidate solution, $p_{1}$ and $p_{2}$ are the selected positions from the memory pool, and N(μ,σ) denotes a normal distribution with mean μ and standard deviation σ.

RDBPSO employs a mini-group processing approach where particles are randomly paired for interaction:

1. Random Permutation: Shuffle particle indices.
2. Pairwise Processing: Process particles in pairs.
3. Performance-Based Roles: Assign “better” and “worse” roles based on fitness comparison.

After each iteration, RDBPSO consolidates all particle memories into a unified pool, *g*_best_ and *g*_second_ are selected by Equation 4.

(4) $$\left\{ \begin{array}{l} M=\{(y_{i},y_{i,\text{second}})|i=1,2,\ldots ,N\}\\ \left(g_{\text{best}},g_{\text{second}})=(M_{\left(1\right)},M_{\left(2\right)}\right) \end{array}\right.$$

where M is the global memory pool containing all particles’ best and second-best positions, N is the number of particles, and $M_{\left(1\right)}$ and $M_{\left(2\right)}$ denote the positions with the first and second lowest fitness values in the sorted memory pool, respectively.

The time complexity of RDBPSO per iteration is *O*(*N* log*N*), while the space complexity is *O*(*N* × *d*). The dropout strategy provides significant computational savings by reducing function evaluations from 4*N* to 2*N* per iteration, achieving 50% efficiency improvement without compromising solution quality.

**Figure 1:**
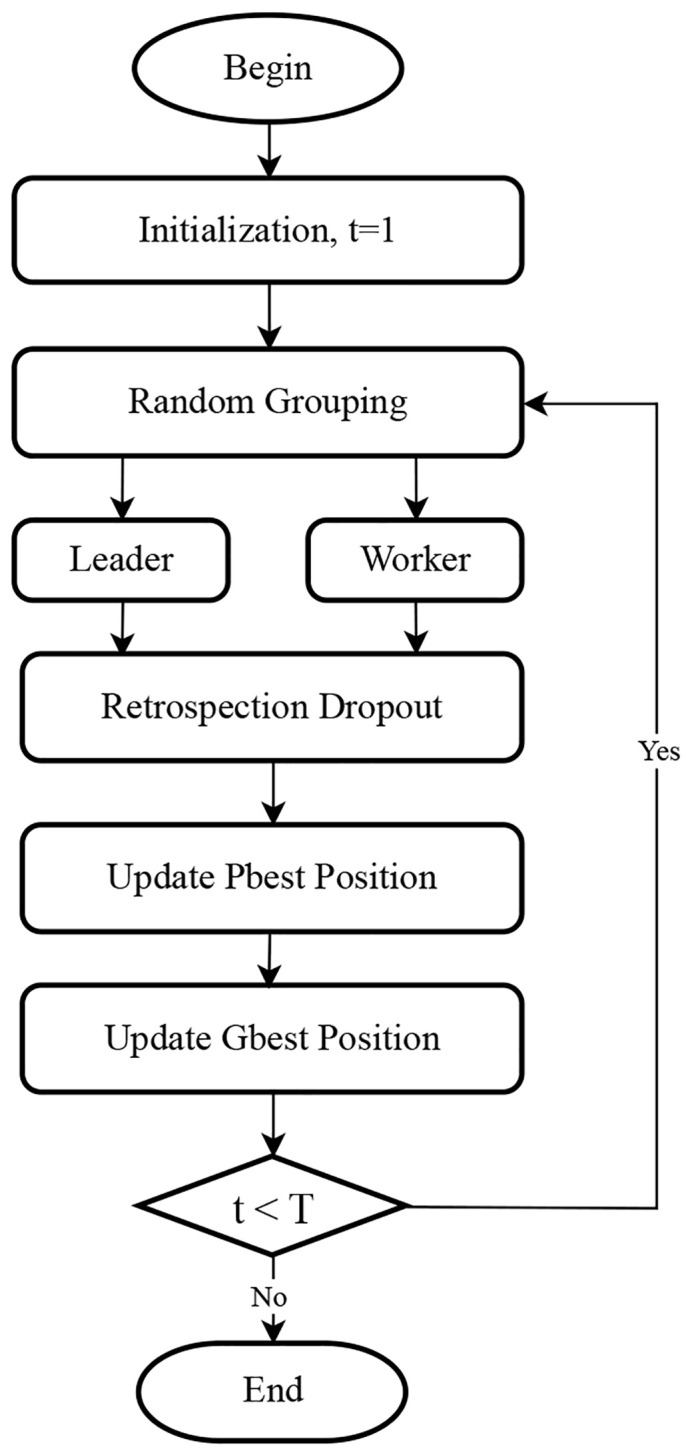
Flowchart for retrospection dropout bare-bones particle swarm optimization.

The flowchart of this task is shown in **Figure 1**.

### 3.3 High-precision classification by RDBPSO

The classification process using RDBPSO follows a systematic approach where medical brain images are transformed into a feature-based optimization problem. In the data preprocessing stage, each brain image is analyzed to extract 16 discriminative features including GLCM-based texture descriptors (contrast, correlation, energy, homogeneity, entropy), statistical measures (mean, standard deviation, skewness, kurtosis, and percentile values q10 and q90), wavelet transform energy coefficients across four sub-bands, and edge density. These features are then standardized to ensure equal contribution across different scales, creating a unified feature space where healthy and tumor samples can be distinguished based on their characteristic patterns.

**Figure 2:**
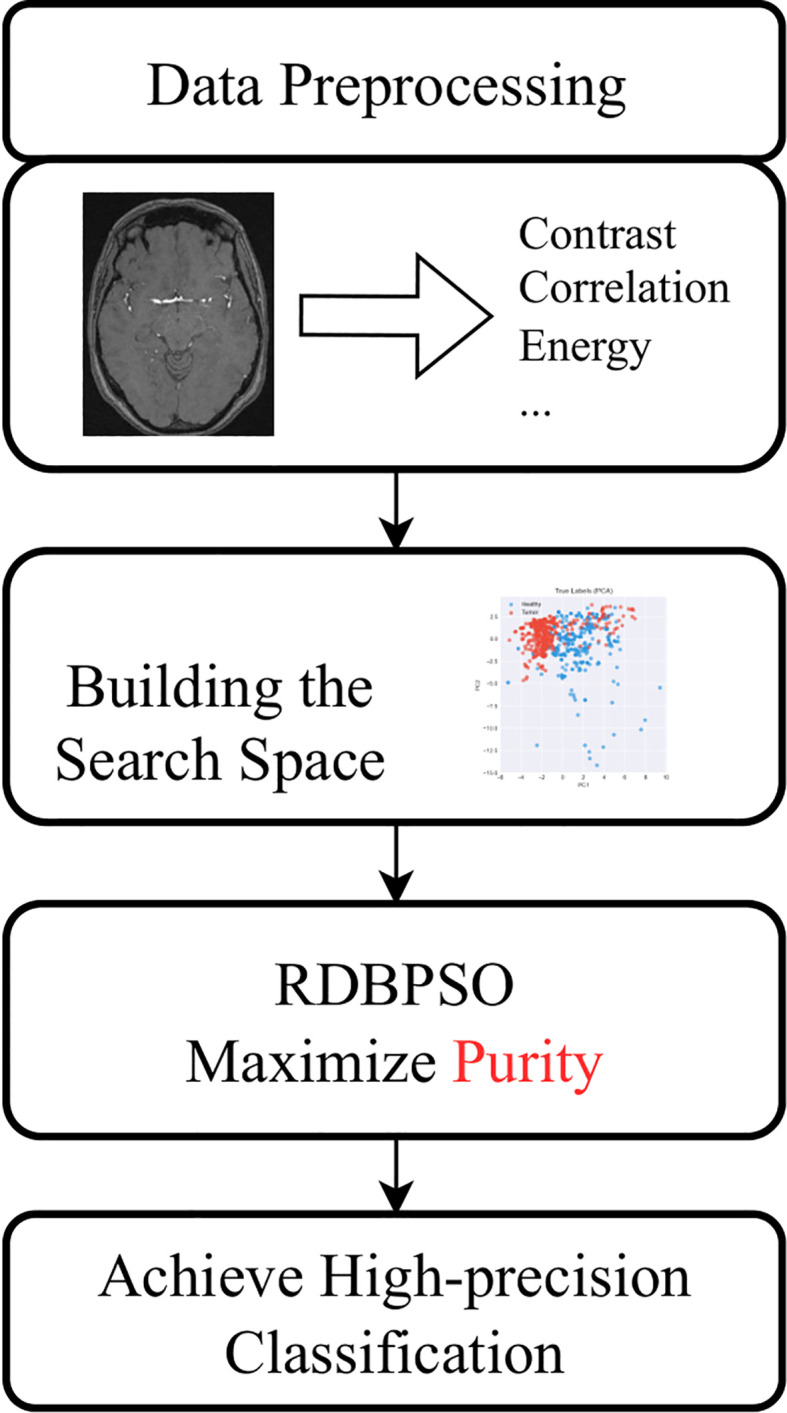
Flowchart for high-precision classification by RDBPSO.

In the RDBPSO optimization framework, each particle represents a complete clustering solution consisting of k cluster centers (k=2 for healthy/tumor classification), encoded as a flattened vector of dimension k×d, where d is the number of features (16 in this case). The objective function maximizes clustering purity, calculated as the sum of the maximum class frequencies within each cluster divided by the total number of samples, effectively measuring how well the clustering separates the two classes. During the search process, particles explore the feature space by generating candidate cluster centers through Gaussian sampling based on combinations of personal and global best positions, with the retrospective dropout mechanism randomly selecting from four possible position pairs to maintain diversity. The optimization continues until convergence, yielding optimal cluster centers that naturally separate healthy and tumor samples in the feature space, enabling rapid classification of new images by simply computing their distances to these learned centers. Details are shown in **Figure 2**.

## 4 Results

### 4.1 Experimental pipeline

To evaluate the effectiveness of the proposed RDBPSO algorithm, we conducted comprehensive comparative experiments with four representative clustering algorithms. Standard PSO serves as the baseline swarm intelligence method. K-means clustering was included as the most widely-used centroid-based algorithm, known for its computational efficiency and simplicity in spherical cluster identification. Hierarchical clustering represents the linkage-based approach, providing a different clustering paradigm that builds clusters through agglomerative merging without requiring prior specification of cluster numbers. Gaussian Mixture Model (GMM) was selected as a probabilistic clustering method that assumes data points are generated from a mixture of Gaussian distributions, offering soft clustering capabilities through probabilistic assignments. All algorithms were configured to identify two clusters corresponding to healthy and tumor cases, with standardized data preprocessing and identical random seeds to ensure fair comparison. The performance evaluation employed five complementary metrics: Accuracy, Adjusted Rand Index (ARI), Normalized Mutual Information (NMI), Fowlkes-Mallows Index (FMI), and Purity, providing a comprehensive assessment of clustering quality from both external validation and cluster cohesion perspectives. For RDBPSO and PSO, population size is 30, max iteration is 100.

**Figure 3:**
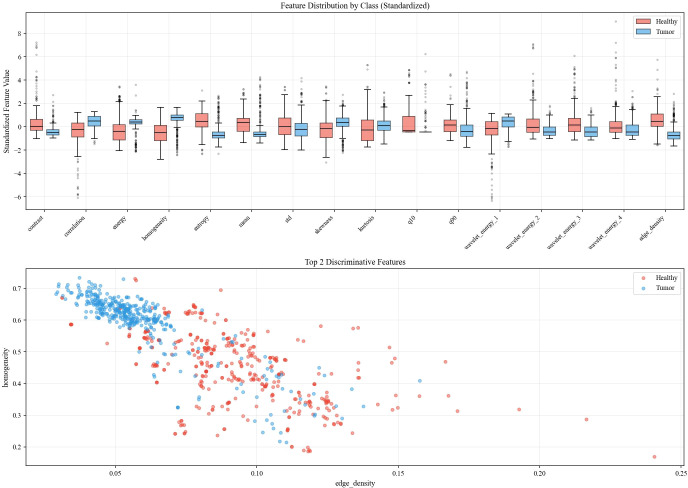
Datasets overview.

**Figure 4:**
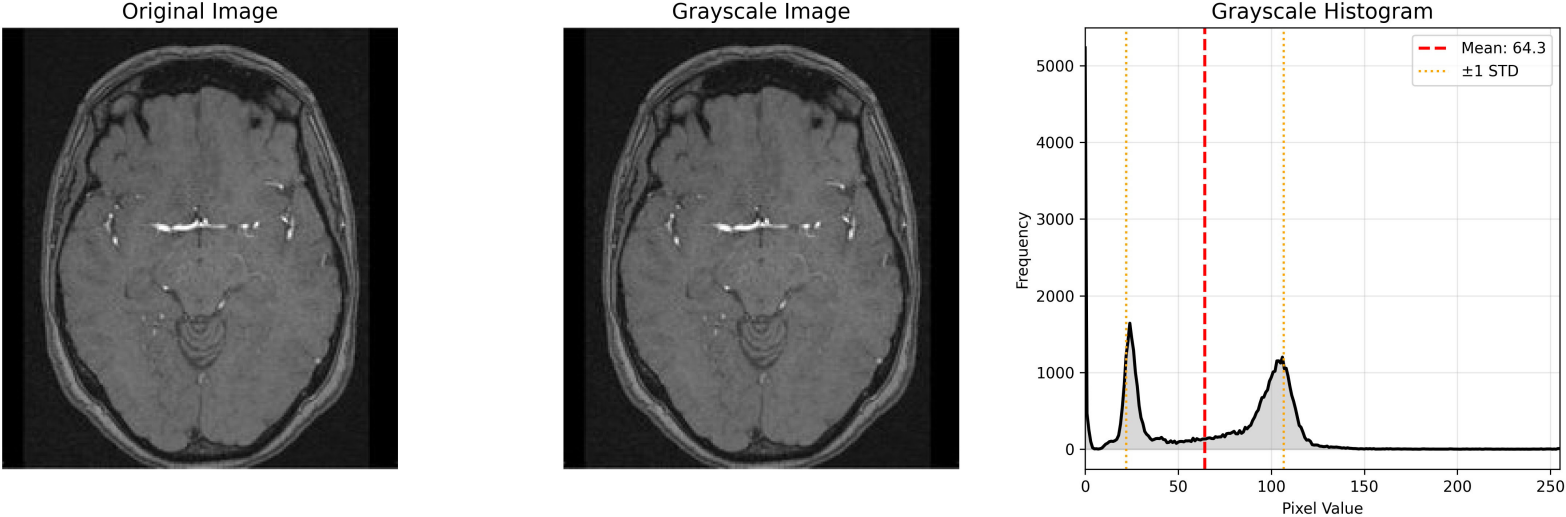
Brain MRI image preprocessing and intensity distribution analysis.

**Figure 5:**
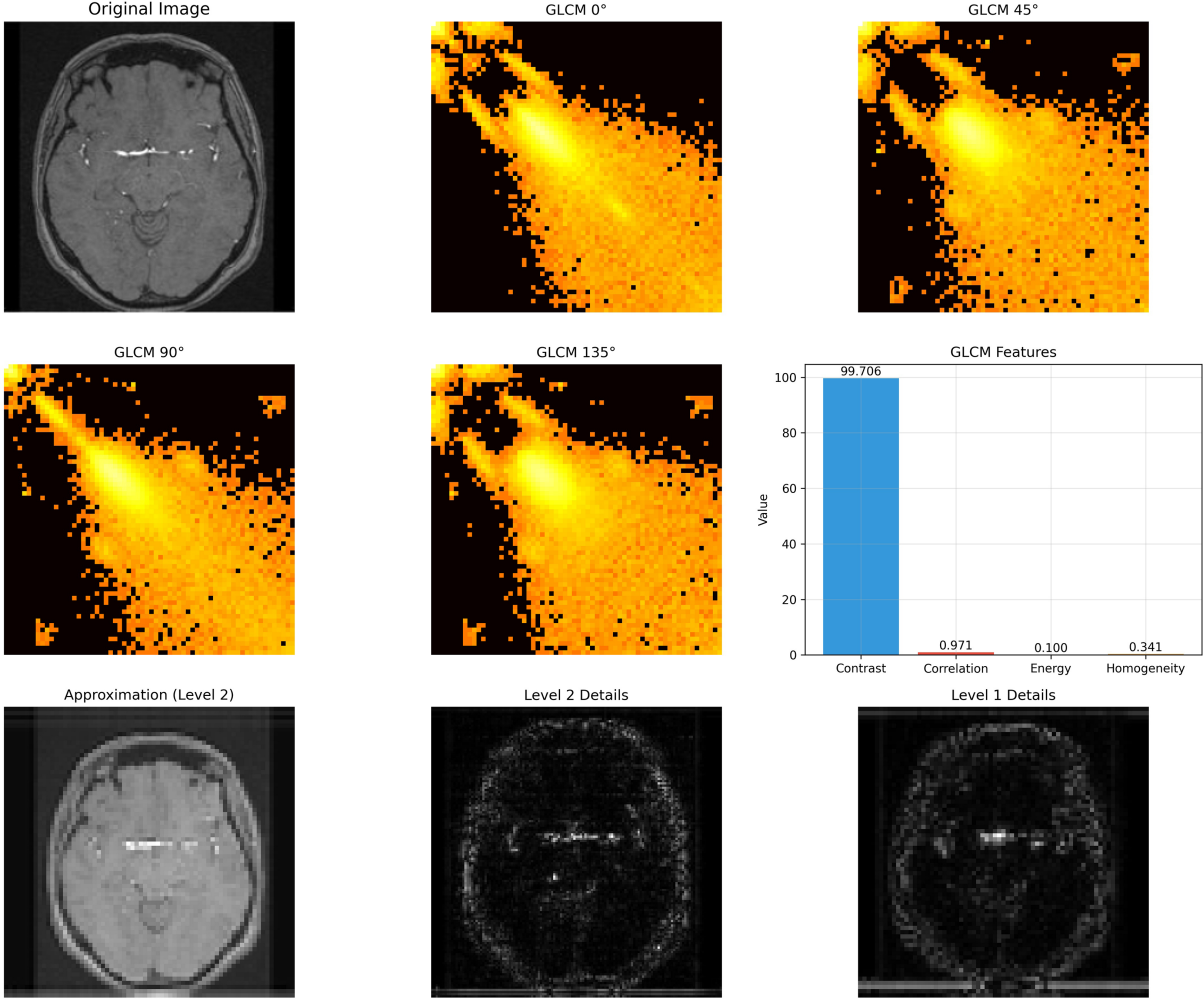
Feature extraction process from brain MRI images using GLCM and wavelet transform.

**Figure 6:**
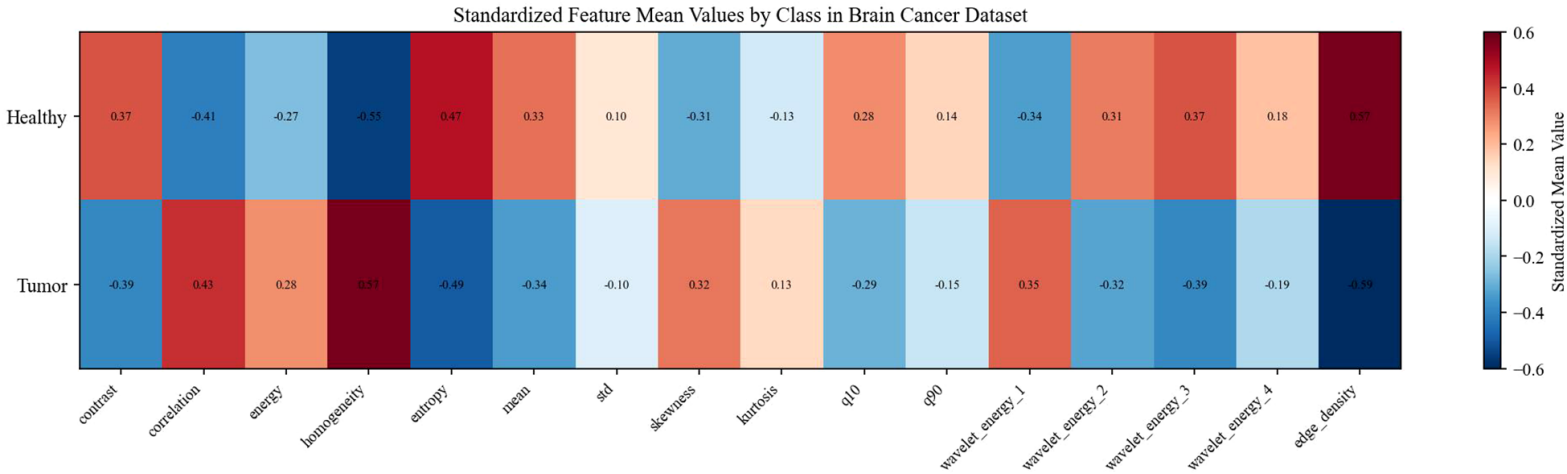
Standardized feature mean values by class in brain cancer dataset.

**Figure 7:**
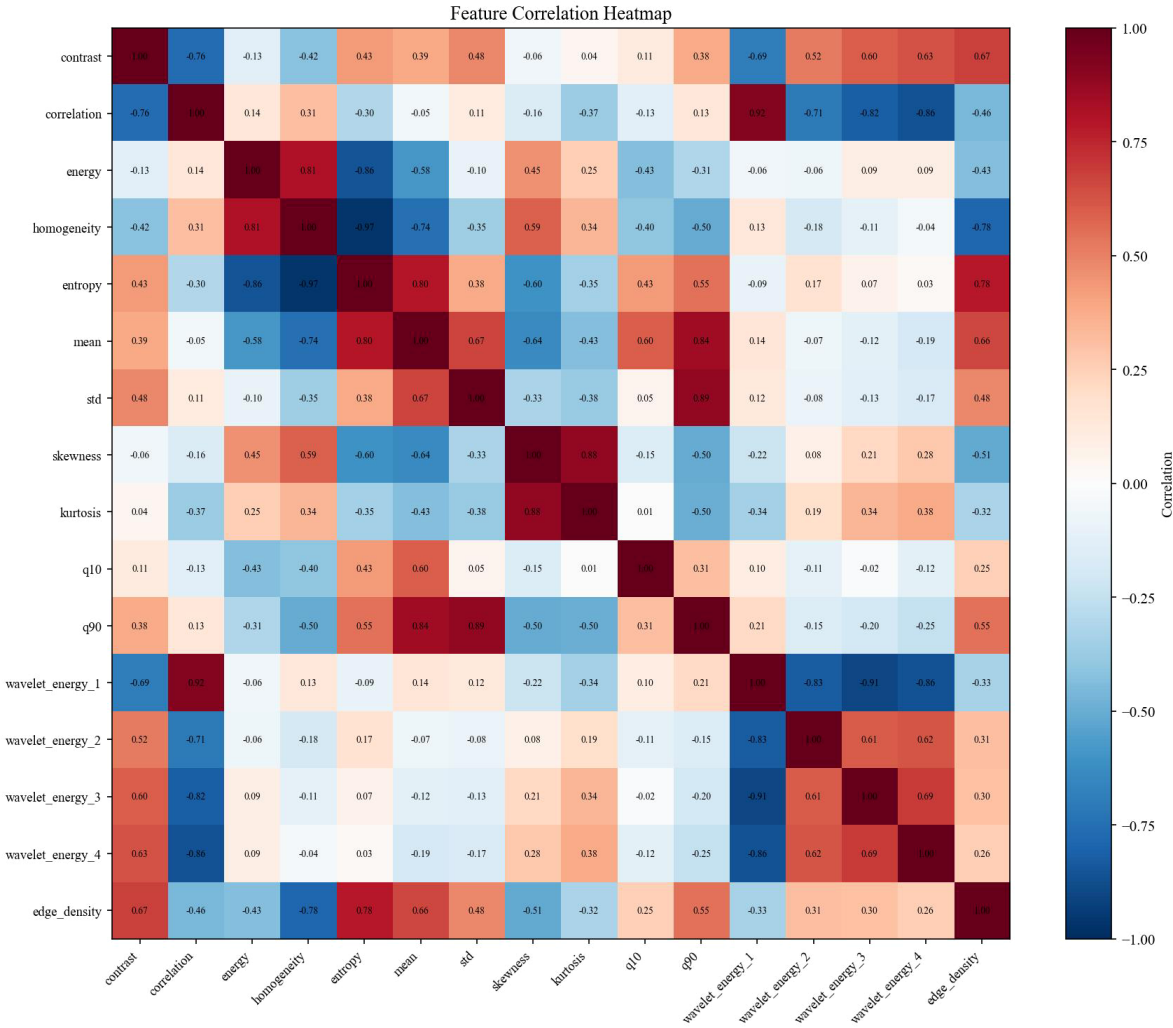
Feature correlation heatmap of brain cancer dataset.

**Figure 8:**
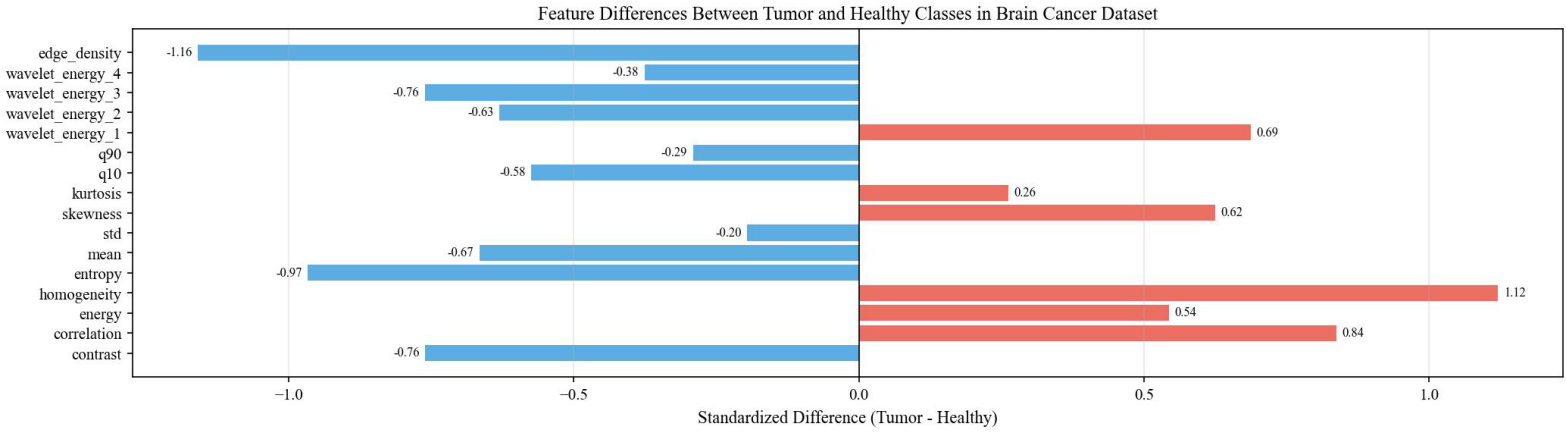
Feature differences between tumor and healthy classes in brain cancer dataset.

The feature analysis reveals distinct discriminative capabilities across the extracted descriptors, with GLCM contrast and grayscale mean exhibiting the most significant differences between healthy and tumor classes, where healthy images demonstrate notably higher values in both metrics. While statistical features such as mean and standard deviation show strong classification potential, more complex texture descriptors including wavelet energy coefficients and edge density display considerable overlap between classes, suggesting limited discriminative power. Interestingly, despite the incorporation of multi-resolution and structural analysis techniques, fundamental statistical features remain the most effective discriminators, with healthy brain images characterized by higher contrast values and elevated grayscale intensities compared to tumor images, which tend to exhibit more concentrated grayscale distributions with lower mean values. This observation underscores the importance of careful feature selection in medical image analysis applications, particularly for rapid screening and classification tasks. Datasets overview is shown in **Figure 3**, details are shown in **Figures 4**–**8**.

The selected feature set represents a comprehensive collection of well-established image descriptors that have demonstrated robust performance across diverse medical imaging applications. GLCM-based texture features constitute fundamental tools in medical image analysis, providing quantitative measures of spatial relationships between pixel intensities that are invariant to linear intensity transformations and capable of capturing subtle textural variations often imperceptible to human observers. These include contrast, correlation, energy, and homogeneity, which collectively characterize the spatial dependency patterns of pixel intensities, alongside entropy, which quantifies the randomness and complexity of the texture distribution. Statistical features, including first-order moments, higher-order moments, and percentile values, serve as essential baseline descriptors that characterize the overall intensity distribution of medical images; specifically, mean, standard deviation, skewness, and kurtosis capture the central tendency, spread, asymmetry, and tail behavior of intensity distributions, while the 10th and 90th percentile values (q10, q90) provide robust boundary estimates less sensitive to outliers. Wavelet transform coefficients enable multi-resolution analysis by decomposing images into frequency-domain components across four sub-bands, allowing simultaneous capture of both spatial and frequency information that is particularly valuable for detecting localized abnormalities at different scales. Edge density provides complementary structural information by quantifying the spatial distribution of intensity gradients within brain tissue regions, reflecting the sharpness and complexity of tissue boundaries that may differ between healthy and tumorous tissues. This multi-faceted approach to feature extraction ensures comprehensive characterization of brain tissue properties from complementary perspectives, creating a robust foundation for automated classification systems that can generalize across different imaging protocols, patient populations, and clinical settings.

### 4.2 Experimental results

The experimental results on the brain cancer dataset demonstrate varying performance levels across the five clustering algorithms, as summarized in **Table 1**.

**Table 1:** Performance comparison of clustering algorithms with rankings.

Algorithm	Accuracy	ARI	NMI	FMI	Purity
RDBPSO	0.9012	0.6436	0.5511	0.8229	0.9012
Standard PSO	0.8925	0.6157	0.5243	0.8093	0.8925
GMM	0.7750	0.3016	0.2410	0.6534	0.7750
K-means	0.7275	0.2061	0.1777	0.6154	0.7275
Hierarchical	0.6388	0.0760	0.0868	0.5860	0.6388

The evaluation of clustering performance employs five complementary metrics that assess different aspects of clustering quality. The mathematical formulations of these metrics are defined in Equation 5:

(5) $$\left\{ \begin{array}{l} \text{Accuracy}=\frac{\sum_{i=1}^{k}\underset{j}{\max}|C_{i}\cap L_{j}|}{n}\\ \text{ARI}=\frac{\text{RI}-E[\text{RI}]}{\text{max }(\text{RI})-E[\text{RI}]},\;\text{where}\;\text{RI}=\frac{a+b}{\left(\begin{array}{c} n\\ 2 \end{array}\right)}\\ \text{NMI}=\frac{2\cdot I(C,L)}{H(C)+H(L)},\;\text{where}\;I(C,L)=\sum_{i=1}^{k}\sum_{j=1}^{l}\frac{\left|C_{i}\cap L_{j}\right|}{n}\text{log }(\frac{n\cdot |C_{i}\cap L_{j}|}{\left|C_{i}|\cdot |L_{j}\right|})\\ \text{FMI}=\frac{TP}{\sqrt{\left(TP+FP)(TP+FN\right)}}\\ \text{Purity}=\frac{1}{n}\sum_{i=1}^{k}\underset{j}{\text{max}}|C_{i}\cap L_{j}| \end{array}\right.$$

where *n* denotes the total number of samples, *k* represents the number of clusters, *l* indicates the number of true classes, $C_{i}$ denotes the *i*-th cluster, and $L_{j}$ represents the *j*-th true class. For ARI calculation, *a* and *b* represent the number of sample pairs that are correctly clustered together and separately, respectively. In the FMI formulation, TP, FP, and FN represent true positives, false positives, and false negatives in terms of sample pair classifications.

RDBPSO achieved the highest performance across all evaluation metrics, with an accuracy of 90.12%, ARI of 0.6436, NMI of 0.5511, FMI of 0.8229, and purity of 90.12%. Standard PSO ranked second with competitive results of 89.25% accuracy, 0.6157 ARI, 0.5243 NMI, 0.8093 FMI, and 89.25% purity, showing substantial improvements over traditional clustering methods while confirming the effectiveness of swarm intelligence approaches for this clustering task. Among the conventional clustering algorithms, GMM performed best with 77.50% accuracy, 0.3016 ARI, 0.2410 NMI, 0.6534 FMI, and 77.50% purity, followed by K-means achieving 72.75% accuracy, 0.2061 ARI, 0.1777 NMI, 0.6154 FMI, and 72.75% purity. Hierarchical clustering showed the lowest performance with 63.88% accuracy, 0.0760 ARI, 0.0868 NMI, 0.5860 FMI, and 63.88% purity. The results reveal a clear performance hierarchy, with swarm intelligence-based methods substantially outperforming traditional clustering approaches, and RDBPSO demonstrating consistent superiority over standard PSO across all evaluation dimensions.

**Figure 9:**
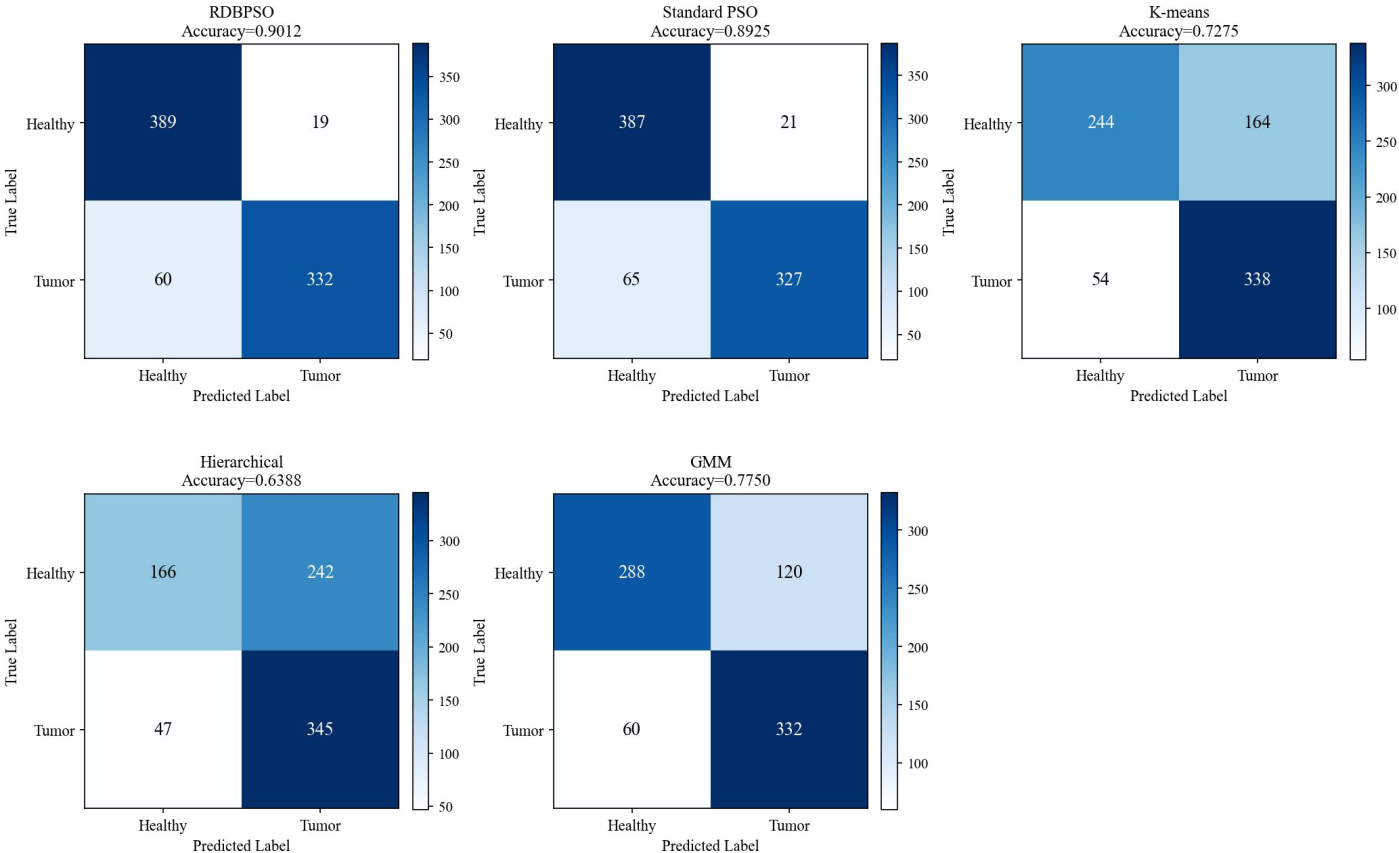
Confusion matrices of five clustering algorithms for brain tumor MRI classification.

**Figure 10:**
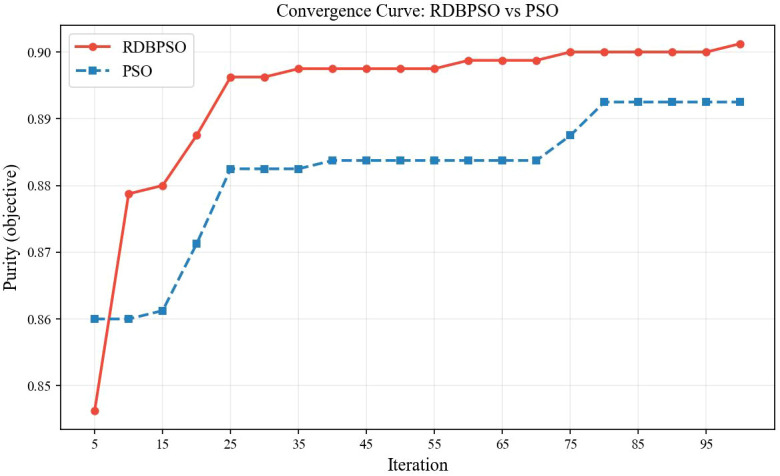
Comparative convergence analysis of retrospection dropout bare-bones PSO and standard pso for medical data clustering.

**Figure 9** presents the confusion matrices of five clustering algorithms applied to the brain cancer MRI dataset, providing a detailed breakdown of classification outcomes for healthy and tumor samples. RDBPSO achieves the best overall performance with 389 true healthy predictions and 332 true tumor predictions, yielding only 19 false tumor assignments and 60 false healthy assignments, corresponding to an accuracy of 90.12%. Standard PSO delivers comparable results with 387 and 327 correct predictions for healthy and tumor classes, respectively, attaining an accuracy of 89.25%. Among the conventional clustering methods, GMM outperforms the remaining two approaches by correctly identifying 288 healthy and 332 tumor samples, while misclassifying 120 healthy samples as tumor and 60 tumor samples as healthy, achieving 77.50% accuracy. K-means demonstrates moderate performance with 244 correct healthy predictions and 338 correct tumor predictions, but exhibits a notably high false negative rate for the healthy class with 164 misclassifications, resulting in 72.75% accuracy. Hierarchical clustering shows the weakest performance, correctly classifying only 166 healthy and 345 tumor samples while generating 242 and 47 erroneous assignments for the respective classes, yielding the lowest accuracy of 63.88%. Collectively, the confusion matrices confirm the consistent superiority of swarm intelligence-based methods, particularly RDBPSO, in maintaining high classification fidelity across both healthy and tumor categories. The convergence behaviors of RDBPSO and PSO algorithms over 100 iterations are shown in **Figure 10**. The experimental results demonstrate that RDBPSO achieves superior convergence speed, greater optimization stability, and higher final solution quality compared to standard PSO, attributable to the synergistic effects of the dual-layer retrospection mechanism and the dropout strategy in preventing premature convergence and sustaining effective exploration throughout the search process.

The experimental validation furnishes compelling empirical evidence substantiating the efficacy of the dual-layer memory architecture and dropout mechanism integrated within the RDBPSO framework. The consistent performance superiority of RDBPSO relative to the standard PSO algorithm across all five evaluation metrics provides conclusive demonstration of the tangible benefits conferred by the retrospective memory strategy. Furthermore, the substantial performance differential observed between swarm intelligence methodologies and conventional clustering approaches—with RDBPSO achieving a 13.88% accuracy improvement over GMM, the most effective traditional method—underscores the fundamental advantages inherent in population-based optimization algorithms equipped with sophisticated memory mechanisms when compared to deterministic clustering paradigms.

### 4.3 Statistical analysis and algorithm stability

**Figure 11:**
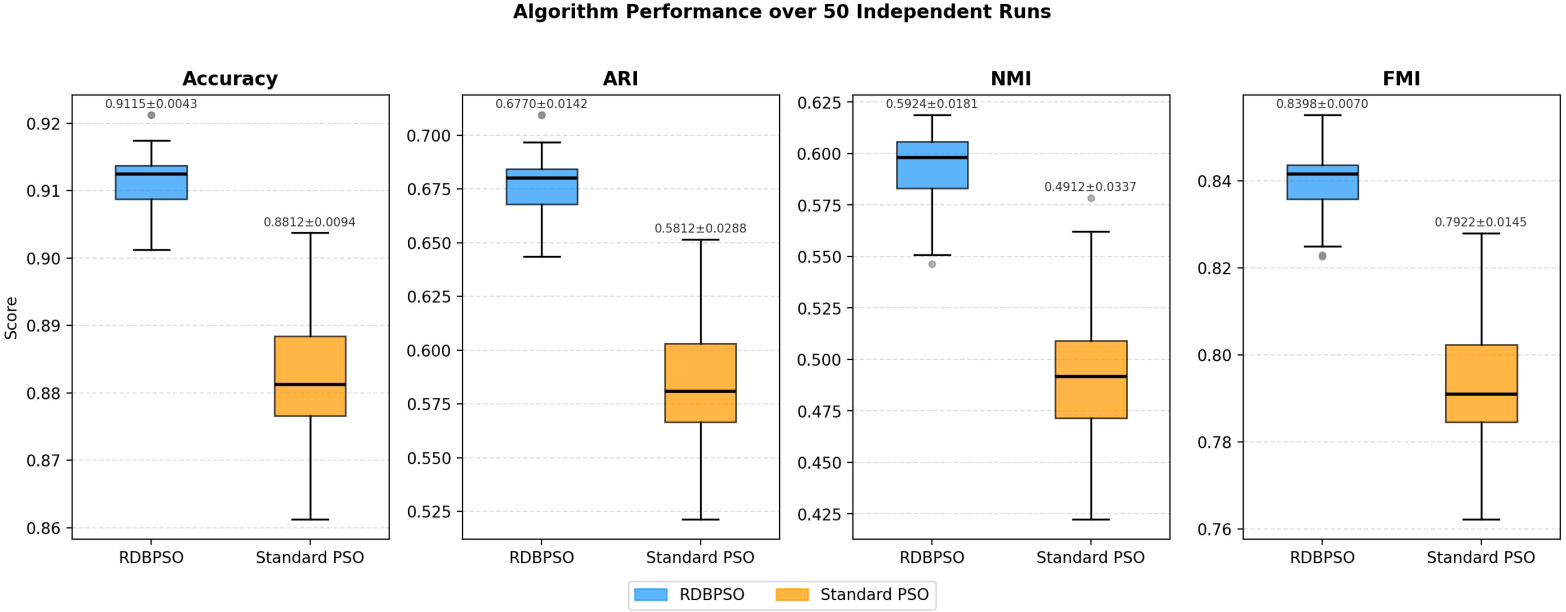
Box plots comparing RDBPSO and Standard PSO across 50 independent runs.

To evaluate algorithmic stability, RDBPSO and Standard PSO were executed across 50 independent runs, using a swarm of 30 particles over 100 iterations. Performance is reported as mean and standard deviation (STD) over 50 runs. Experimental results are shown in **Table 2**, and box plots are shown in **Figure 11**. RDBPSO consistently outperformed Standard PSO on all metrics while exhibiting substantially lower variance, indicating that its superior performance is not attributable to favorable random initialization but reflective of a genuine algorithmic advantage. In particular, the standard deviation of RDBPSO across all metrics remained notably small, suggesting that the algorithm converges reliably to high-quality solutions regardless of the starting conditions. These findings collectively demonstrate that RDBPSO achieves both higher clustering accuracy and greater stability, making it a more dependable choice for brain cancer subtype identification in practical applications.

**Table 2:** Performance comparison of RDBPSO and standard PSO over 50 independent runs.

Algorithm		Accuracy	ARI	NMI	FMI
RDBPSO	Mean Std	**0.9115** (0.0043)	**0.6770** (0.0142)	**0.5924** (0.0181)	**0.8398** (0.0070)
Standard PSO	Mean Std	0.8812 (0.0094)	0.5812 (0.0288)	0.4912 (0.0337)	0.7922 (0.0145)

Bold indicates the better result per metric.

## 5 Discussion

The superior performance of RDBPSO can be attributed to several key algorithmic innovations that address fundamental limitations of traditional optimization approaches. The retrospection mechanism enables particles to maintain dual-layer memory of both best and second-best positions, providing enhanced exploration capabilities when the current search direction becomes ineffective. This memory diversity proves particularly valuable in the complex, multi-modal fitness landscape of clustering optimization, where premature convergence to local optima frequently occurs in standard PSO. The dropout strategy, which randomly selects only two out of four possible position combinations during candidate generation, introduces controlled stochasticity that prevents algorithmic stagnation while maintaining computational efficiency. The substantial performance gap between RDBPSO and standard PSO demonstrates that these enhancements provide meaningful improvements beyond simple parameter tuning. The consistently poor performance of traditional clustering methods highlights the inherent challenges of the brain cancer classification task, where the high-dimensional feature space and potential non-linear cluster boundaries make conventional distance-based approaches inadequate. Notably, the strong correlation between accuracy and purity metrics confirms that the clustering task exhibits clear class separability when appropriate optimization techniques are employed. The moderate ARI values suggest that while the proposed method achieves excellent classification accuracy, there remains room for improvement in cluster structure optimization, potentially through incorporating additional clustering validity indices into the objective function.

**Figure 12:**
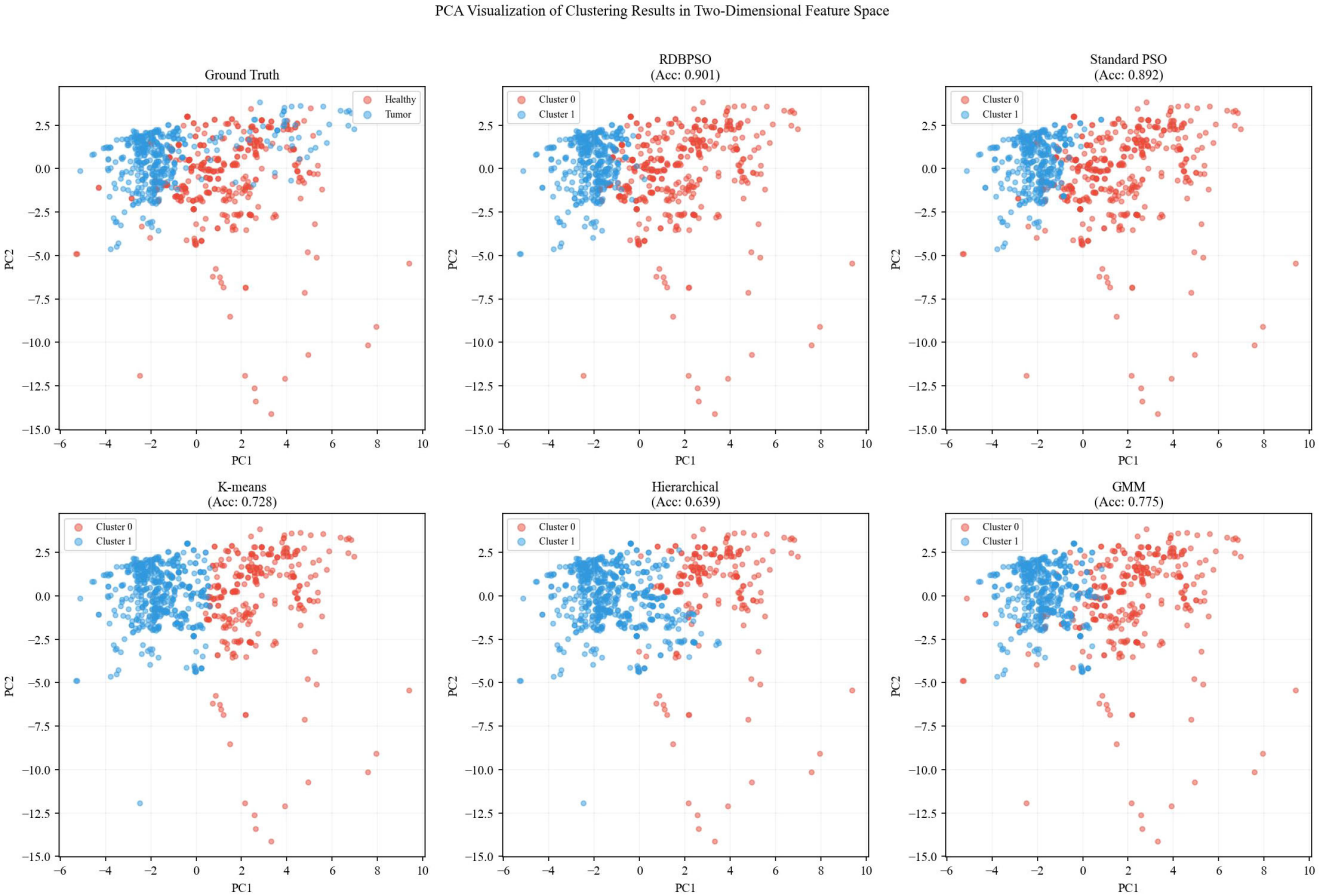
PCA visualization of clustering results in two-dimensional feature space.

**Figure 12** shows the PCA (Principal Component Analysis) visualization of the brain cancer clustering results, where the high-dimensional feature data is projected onto a two-dimensional space defined by the first two principal components (PC1 and PC2). The Ground Truth subplot displays the actual class labels (healthy vs. tumor), while the remaining five subplots present the clustering results from different algorithms, with red and light blue points representing the two identified clusters (Cluster 0 and Cluster 1, respectively).

**Figure 13:**
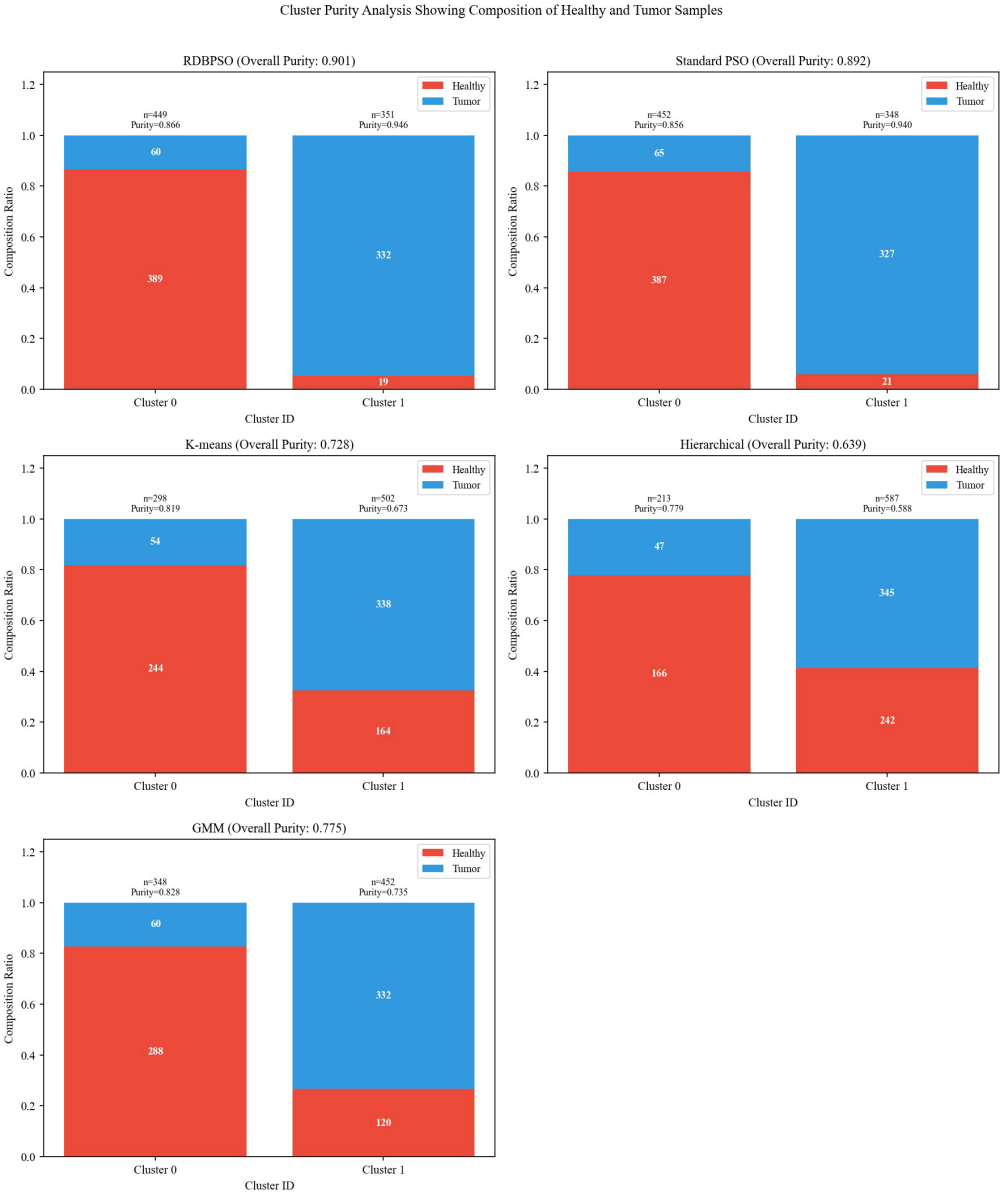
Cluster purity analysis showing composition of health and tumor samples.

**Figure 13** illustrates the clustering purity analysis for each algorithm, where stacked bar charts display the composition ratio of healthy (red) and tumor (light blue) samples within each identified cluster, with cluster size and individual purity values annotated above each bar.

### 5.1 Limitation and future work

Despite the encouraging results demonstrated by the proposed RDBPSO algorithm, several limitations merit careful consideration. First, the experimental validation was conducted exclusively on a single publicly available brain MRI datasets, which may constrain the generalizability of the findings across heterogeneous imaging protocols, diverse scanner configurations, and varying patient demographics. Second, the present study is confined to binary classification between healthy and tumorous tissues; the scalability and efficacy of RDBPSO in multi-class tumor subtype classification scenarios, such as the simultaneous differentiation of glioma, meningioma, and pituitary tumors, remain to be rigorously investigated. Third, the dropout mechanism introduces additional hyperparameters, notably the selection ratio governing particle interaction combinations, whose optimal configuration is likely to vary considerably across different datasets and problem domains, thereby necessitating systematic and task-specific hyperparameter tuning in practical deployment. Fourth, the current implementation relies upon a fixed dual-layer memory structure, which may not constitute a universally optimal design; adaptive memory depth mechanisms capable of dynamically adjusting historical solution retention in accordance with real-time convergence behavior represent a promising avenue for further algorithmic refinement and performance enhancement. Fifth, it should be acknowledged that clustering purity serves as both the optimization objective and one of the evaluation metrics in this study. This objective-evaluation coupling may introduce a potential bias, whereby the algorithm is implicitly optimized toward performing well on this specific metric, which could inflate the apparent advantage of RDBPSO relative to non-purity-optimized methods on this particular measure. Future work should consider adopting an optimization objective that is decoupled from the evaluation metrics, to provide a more unbiased assessment of algorithmic performance.

A promising future research direction is to utilize synthetic data generation techniques to augment existing training data, thereby addressing the inherent scarcity of annotated medical image datasets. Applying the review mechanism and dropout strategy developed in this study to the synthetic data generation process to improve its accuracy and diversity is an important and valuable direction for future research. Also, investigating adaptive dropout probabilities that automatically regulate the particle interaction selection ratio in accordance with population diversity metrics may further optimize the balance between exploration and exploitation throughout the optimization process. Third, evaluating RDBPSO’s scalability on larger and more heterogeneous datasets will be essential for establishing its generalizability across diverse clinical environments and heterogeneous imaging protocols.

## 6 Conclusion

This paper proposed RDBPSO (Retrospection Dropout Bare-Bones Particle Swarm Optimization), a novel swarm intelligence-based clustering algorithm that incorporates retrospective memory mechanisms and strategic dropout techniques for enhanced optimization performance. The retrospection mechanism maintains dual-layer memory of both best and second-best positions for each particle, enabling effective exploration when current search directions become suboptimal. The dropout strategy randomly selects two out of four possible position combinations during candidate generation, introducing controlled stochastic-city that prevents algorithmic stagnation while maintaining computational efficiency. These innovations collectively address the premature convergence and local optima problems inherent in traditional particle swarm optimization approaches, particularly in complex multi-modal clustering optimization landscapes.

Comprehensive experimental validation on a brain cancer dataset demonstrated the superior performance of RDBPSO in complex classification tasks. The proposed algorithm achieved exceptional results. The results confirm that RDBPSO effectively handles the high-dimensional feature space and complex cluster boundaries characteristic of biomedical image data, demonstrating its algorithmic potential as an annotation-efficient framework for brain tumor classification, with future work needed to assess its applicability on more diverse and clinically representative datasets.

Future work will focus on several promising directions to further enhance the algorithm’s capabilities and applicability. First, extending the retrospection mechanism to maintain longer historical memories could provide richer guidance for particle movement in complex search spaces. Second, investigating adaptive dropout probabilities that dynamically adjust based on convergence behavior may optimize the balance between exploration and exploitation. Third, evaluating RDBPSO’s scalability on larger datasets with varying cluster numbers will assess its generalizability across different problem scales. Additionally, incorporating multi-objective optimization frameworks could enable simultaneous optimization of multiple clustering criteria, potentially leading to more robust solutions for diverse biomedical clustering applications beyond cancer classification.

## Data Availability

The original contributions presented in the study are included in the article/supplementary material. Further inquiries can be directed to the corresponding authors.
